# Supplementary material for: Iron Oxide Nanoparticles for Visualization of Prostate Cancer in MRI
Source: Cancers (Basel). 2022 Jun 13;14(12):2909. doi: 10.3390/cancers14122909 (PMC9221397; doi:10.3390/cancers14122909)
Supplement: Supplementary file 1 [file cancers-14-02909-s001.zip › cancers-1702238-supplementary.pdf]

cancers

(A)

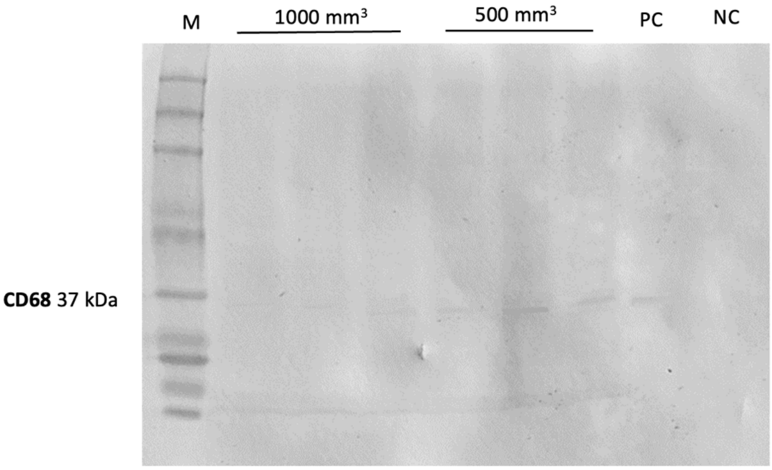

(B)

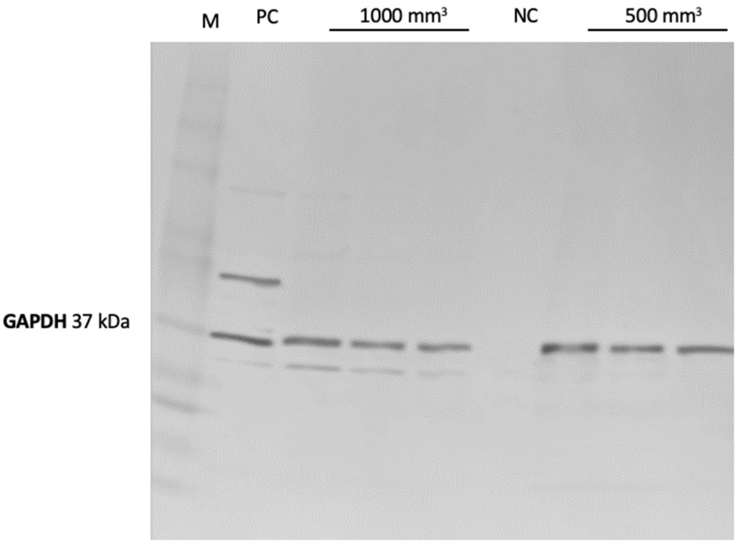

Figure S1. Western Blot analyse. A: Western Blot analyse for CD68. For each group, 3 tumors (n = 3 per group) were used for western blot analysis to detect the expression of CD68. Relative intensity from left to right: 290.1; 321.6; 302.7; 618; 966.0; 1144.4; PC: 1174.2. **B:** Western Blot analyse for GAPDH. For each group, 3 tumors (n = 3 per group) were used for western blot analysis to control protein levels.
